# Cold-induced urticarial autoinflammatory syndrome related to factor XII activation

**DOI:** 10.1038/s41467-019-13984-8

**Published:** 2020-01-10

**Authors:** Jörg Scheffel, Niklas A. Mahnke, Zonne L. M. Hofman, Steven de Maat, Jim Wu, Hanna Bonnekoh, Reuben J. Pengelly, Sarah Ennis, John W. Holloway, Marieluise Kirchner, Philipp Mertins, Martin K. Church, Marcus Maurer, Coen Maas, Karoline Krause

**Affiliations:** 1 Department of Dermatology and Allergy, Charité — Universitätsmedizin Berlin, Charitéplatz 1, 10117 Berlin, Germany; 2 Autoinflammation Reference Center Charité (ARC²), Charité — Universitätsmedizin Berlin, Charitéplatz 1, Berlin, 10117 Germany; 3 University Medical Center Utrecht, Department of Clinical Chemistry and Haematology, Heidelberglaan 100, 3584 CX Utrecht, The Netherlands; 4 Human Genetics & Genomic Medicine, Faculty of Medicine, University of Southampton, University Hospitals Southampton, Southampton, SO16 6YD Hampshire, UK; 5 Proteomics Platform, Berlin Institute of Health (BIH) and Max Delbrück Center for Molecular Medicine in the Helmholtz Association (MDC), 13125 Berlin, Germany

**Keywords:** Medical genetics, Coagulation system, Immunogenetics, Autoinflammatory syndrome

## Abstract

Hereditary autoinflammatory diseases are caused by gene mutations of the innate immune pathway, e.g. nucleotide receptor protein 3 (NLRP3). Here, we report a four-generation family with cold-induced urticarial rash, arthralgia, chills, headache and malaise associated with an autosomal-dominant inheritance. Genetic studies identify a substitution mutation in gene *F12* (*T859A*, resulting in p.*W268R*) which encodes coagulation factor XII (FXII). Functional analysis reveals enhanced autocatalytic cleavage of the mutated protein and spontaneous FXII activation in patient plasma and in supernatant of transfected HEK293 cells expressing recombinant W268R-mutated proteins. Furthermore, we observe reduced plasma prekallikrein, cleaved high molecular weight kininogen and elevated plasma bradykinin. Neutrophils are identified as a local source of FXII. Interleukin-1β (IL-1β) is upregulated in lesional skin and mononuclear donor cells exposed to recombinant mutant proteins. Treatment with icatibant (bradykinin-B2-antagonist) or anakinra (interleukin-1-antagonist) reduces disease activity in patients. In conclusion, our findings provide a link between contact system activation and cytokine-mediated inflammation.

## Abstract (web summary)

Systemic autoinflammatory syndromes such as cryopyrin-associated periodic syndrome (CAPS) are rare and often involve genes related to the inflammasome. Here, the authors report a syndrome characterised by systemic inflammation and cold-induced urticarial rash associated with a Factor XII-activating mutation.

## Introduction

Systemic autoinflammatory diseases (SAIDs) are characterized by self-directed inflammation that is mediated via activation of innate immune pathways. The majority of SAIDs are rare and disabling disorders with significant morbidity and quality of life impairment. Within the last 15 years, the molecular basis of several hereditary SAIDs was uncovered by using novel high-throughput sequencing techniques. Many of the affected genes were shown to involve molecules associated with inflammasome signaling. Inflammasomes are intracellular multiprotein complexes, which assemble upon recognition of diverse pathogen- and danger-associated molecular patterns (PAMPs/DAMPs) leading to activation of caspase-1 to convert pro-interleukin-1β (pro-IL-1β) and pro-IL-18 into their active forms. Consequently, cytokine-targeted therapies, in particular directed against IL-1β and its receptor, largely improved the treatment of SAID patients.

Cryopyrin-associated periodic syndrome (CAPS) is one of the best-studied hereditary SAIDs. Its hallmark feature is recurrent urticarial rash combined with systemic symptoms, such as fever episodes, arthropathy, uveitis, headaches, and malaise. Cold temperatures represent a major trigger of the disease. CAPS is caused by a heterozygous mutation in the gene encoding for nucleotide binding like receptor protein 3 (NLRP3 or cryopyrin)^1^. The *NLRP3* mutation in CAPS results in an overactivated inflammasome followed by IL-1β-mediated inflammation. A diagnosis of CAPS is often delayed, and only eventually made by excluding other immune-mediated disorders, such as infections, immunodeficiencies, autoimmune, and neoplastic diseases. Previously, up to 40% of cases with typical CAPS phenotype were reported to lack disease-causing *NLRP3* mutations, suggesting the existence of other, as of yet unknown genetic variants^2^. The increased use of sequencing techniques identified a number of somatic CAPS mutations in patients tested negative by conventional Sanger sequencing (depending on the study and phenotype in 12–69% of individuals)^3,4^. Besides CAPS, few other genetic defects were found, including mutations in *NLRP12, NLRC4*, and *PLCG2*. All of them present with early onset urticarial rash triggered by cold and an autoinflammatory phenotype^5–9^.

Here, we describe an autosomal-dominant disease characterized by cold-induced urticarial rash and systemic inflammation. The disease is associated with a mutation in the human coagulation Factor 12 gene (*F12*), and we propose the term FXII-associated cold autoinflammatory syndrome (FACAS) to denote this disease. Functional studies show autocatalytic cleavage of FXII and profound activation of the contact system linked to IL-1β-mediated inflammation.

## Results

### Clinical phenotype and disease manifestation

**Fig. 1:**
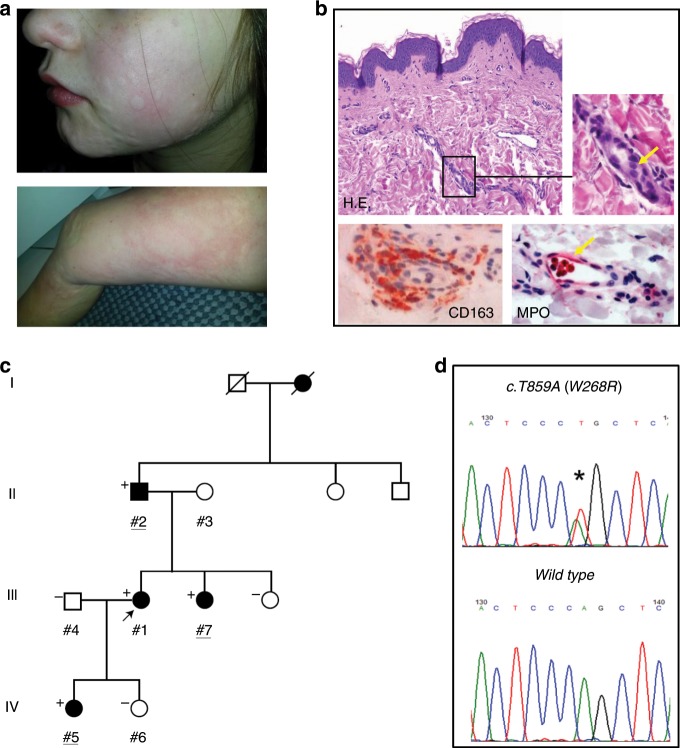
Phenotype and genotype in patients with FXII-associated cold autoinflammatory syndrome (FACAS). **a** Urticarial rash of the face of a 9-year-old girl and the leg of her 35-year-old mother. **b** Skin histopathology reveals dermal edema (H.E. yellow arrow) and perivascular infiltrates composed of macrophages (CD163) and few intravascular neutrophils (myeloperoxidase [MPO], yellow arrow). Original magnification ×400  (*n* = 2 technical replicates). **c** Family pedigree over four generations identifying affected (shaded black) and unaffected (unshaded) individuals. Exome sequenced individuals are underlined. Where conducted, the results of Sanger sequencing of p.W286R are shown to top left of each individual whereby “+” denotes heterozygote for variant and wild-type, and “−” denotes healthy reference. **d** Representative sequencing chromatogram of the heterozygous genotype in an affected individual (top) and chromatogram of healthy individual from the same region (bottom).

Four family members of a four-generation family of European ancestry presented with cold-induced urticarial rash that started within the first weeks after birth (Table 1; Fig. 1a). The non-pruritic burning rash occurred on a daily basis at the extremities, face, and trunk, and was not accompanied by angioedema. Wheal development typically appeared within 10–30 min after whole-body exposure to cold temperatures (<15–20 °C) and stayed for one-to-several hours after rewarming. The urticarial rash was exacerbated by windy and humid weather. Wheals were induced by sweating and consecutive evaporative cooling. Direct localized skin contact with cold fluids or objects, however, did not cause any symptoms. The skin symptoms associated with signs of systemic inflammation including chills, joint pain, headache, fatigue, and subfebrile evening temperatures. All patients had perennial symptoms that deteriorated during the cold season and frequently lead to absence from school and work. Over the course of their disease, patients reported worsening of systemic manifestations, e.g., joint pain, fatigue, and chills, resulting in a more severe phenotype in adult patients. The clinical signs were combined with mild-to-moderate increases in serum levels of the inflammation markers amyloid A (SAA) and S100 A12/S100 A8/9, but not C-reactive protein (CRP). Other routine laboratory markers (e.g., liver and kidney function, blood count, coagulation) were unremarkable. Histopathology of lesional skin following cold exposure revealed moderate dermal edema and perivascular infiltrates composed of macrophages and few neutrophils (Fig. 1b). In contrast to acquired cold urticaria, affected individuals presented with generalized urticarial rash upon cold exposure and tested negative in the ice cube skin provocation test. Cold water bath provocation of the forearm (15 °C for 10 min) did also not cause any wheals or angioedema. In addition, all patients reported complete unresponsiveness to oral antihistamines at standard or higher than standard doses (please see Supplementary Table 1 for a comparison of FACAS with other cold-induced urticarial disorders). A therapeutic trial with IL-1 receptor antagonist anakinra in patients #1 and #2 resulted only in limited (#2) to moderate improvement (#1) of clinical symptoms. However, continuous anti-IL-1 treatment could not be maintained due to severe injection site reactions.

**Table 1:** Individual clinical characteristics and manifestations in FACAS patients.

Characteristic	Patient ID #1	Patient ID #2	Patient ID #5	Patient ID #7
Age at diagnosis	35	64	9	27
Sex	Female	Male	Female	Female
Age at disease onset	After birth	After birth	After birth	After birth
Skin symptoms	Cold-induced urticarial rash	Cold-induced urticarial rash	Cold-induced urticarial rash	Cold-induced urticarial rash
CNS symptoms	Headache	Headache	Headache	Headache
Musculo-skeletal symptoms	Moderate arthralgia	Severe arthralgia	None	Mild arthralgia
Systemic symptoms	Fatigue, subfebrile temperatures	Fatigue, subfebrile temperatures	Fatigue	Fatigue
S100 A12 in ng/ml (ref. <200)	523.7	n.a.	1024.3	n.a.
S100 A8/9 in µg/ml (ref. <2.94)	n.a.	4.10	n.a.	4.51
Serum amyloid A in mg/l (ref. <6.40)	16.80	n.a.	n.a.	8.20

### Genetic analysis

Based on the clinical phenotype resembling CAPS, Sanger sequencing of *NLRP3* and *NLRP12* was initially performed, but failed to identify plausible pathogenic mutations in the index patient (#1). The following whole-exome sequencing prioritized the assessment of genes that were previously associated with a skin phenotype of urticaria or urticaria-like symptoms as reported by the Human Gene Mutation Database (Release 2015.4)^10^. These were *TBXA2R*, *CEP68*, *F12*, *SERPING1*, *NLRP3*, *NLRP12*, *KIT*, *IL18*, *HNMT*, *FCER1α*, *NLRC4*, *ADGRE2*, and *PLCG2*. Sequencing data were generated for three affected family members across three generations (#2, #5, #7, Fig. 1c) and filtered to identify a single candidate variant, a heterozygous substitution mutation in exon 9 of *F12* (Fig. 1d*)*, the gene encoding for the human coagulation factor XII (NM_000505:*c.T859A:p.W268R*). The observed variant is not listed in the Genome Aggregation Consortium (gnomAD) database listing ~140,000 individuals^11^. In silico predictors of variant pathogenicity concordantly inferred deleterious impact (GERP = 5.01; SIFT = 0.0; PolyPhen2 = 1.0). Sanger sequencing of the available family members confirmed consistent segregation of this mutation with the prior annotation of disease status (Fig. 1c, d).

FXII exerts diverse functions in coagulation, fibrinolysis, complement activation, and the kallikrein–kinin system. Mutations in *F12* are linked to hereditary angioedema with normal C1 inhibitor (FXII-HAE)^12^. FXII-HAE is characterized by acute attacks of localized tissue swellings that are mediated by the vasoactive peptide bradykinin, which is produced by the plasma kallikrein–kinin system. Known FXII-HAE mutations are primarily located in the proline-rich region on exon 9^13,14^. In contrast, the W268R variant lies within the triple-looped kringle domain of FXII.

### Functional analyses

Markers of coagulation (activated partial thromboplastin time [aPTT], fibrinogen, plasminogen, FXI activity, D-dimers, INR) and complement activation (C3, C4, C1-esterase inhibitor [C1-INH] concentration and function) were normal in all affected family members.

**Fig. 2:**
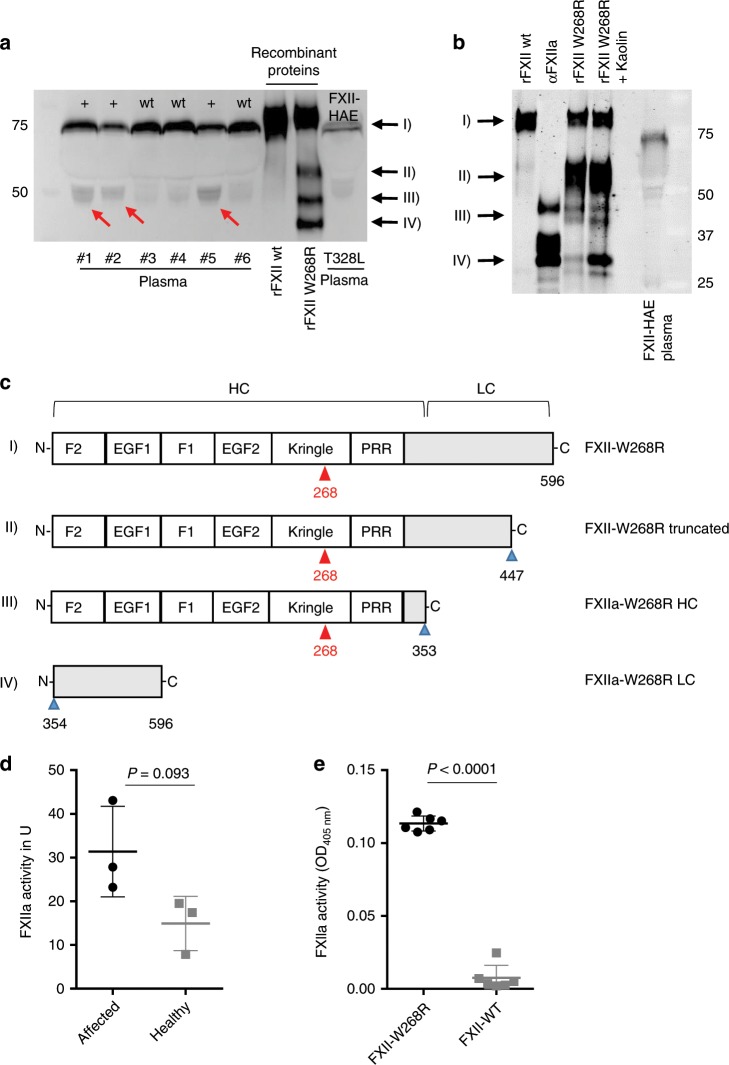
FXII W268R results in fragmentation and spontaneous activation of FXII. **a** FXII fragmentation in FACAS plasma samples and FXII W268R mutant proteins. Citrate plasma of affected (#1, #2, #5) and non-affected (#3, #4, #6) family members, recombinant wild-type and W268R FXII expressed in HEK293 cells and plasma from an FXII-HAE patient was separated by SDS-PAGE (reducing blot) and immunoblotted for FXII. Red arrows at 50 kDa indicate heavy chain of cleaved FXII in the plasma of affected patients and recombinant W268R FXII (rFXII W268R), which is barely visible in non-affected and hereditary angioedema (HAE) III plasma or wild-type FXII (rFXII wt). Recombinant W268R and wild-type FXII were expressed in HEK293 cells. Black arrows point to rFXII W268R fragments observed by mass spectrometry in **c**, *n* = 5 technical replicates. **b** Immunoblot (reduced conditions) of recombinant mutant proteins (in the presence or absence of surface activator kaolin) and controls (rFXII wt and purified αFXIIa). In addition to 50 kDa (heavy chain, HC) and 25 kDa (light chain, LC) bands, a further band appears at 60 kDa (II) that is absent in rFXII wt and αFXIIa. Black arrows point to rFXII W268R fragments observed by mass spectrometry in **c**, *n* = 3 technical replicates. **c** Schematic presentation of FXII W268R procession. Red arrows point to mutation at position W268R. Proteolytic sites are indicated by blue arrows. Cleavage after position 353 results in FXIIa W268R HC (50 kDa) and FXIIa W268R LC (25 kDa) fragments. Additional cleavage results in a truncated FXII W268R fragment (60 kDa) cleaved at position 447. **d** FXIIa activity was measured by a chromogenic assay demonstrating considerable spontaneous FXIIa activity in plasma of affected individuals (*n* = 3), but less in healthy donors (*n* = 3 healthy family members). **e** Spontaneous FXIIa activity was also observed in culture supernatants from HEK293 cells expressing W268R, but not wild-type FXII. Graphs show individual data points and the mean ± s.e.m. Statistical significance is indicated by unpaired Student’s *t* test with Welch’s correction (**d**, **e**). Source data are provided as a source data file.

To test, if the FXII-*p.W268R* substitution in our patients leads to cleavage and/or activation of FXII associated with contact system activation and subsequent generation of bradykinin, we first assessed FXII fragmentation and activity. For its activation, FXII needs to be cleaved into a two-chain molecule composed of a heavy- and a light-chain linked by a disulfide bond^15^. In addition to the unprocessed FXII (78 kDa), molecular analyses revealed an increased presence of the 50 kDa band (reducing) in the FXII immunoblot from plasma of affected family members, which was hardly detectable in healthy control family members and an FXII-HAE control (Fig. 2a). This band resembled the heavy chain of active FXII (FXIIa), suggesting partial fragmentation and activation of FXII in affected patients. Accordingly, recombinant FXII-*p.W268R* (rFXII W268R) expressed in HEK293 cells also showed the characteristic 50 kDa (III) band in the FXII immunoblot resembling the heavy chain. This band is also present in purified αFXIIa, but not found in unprocessed recombinant FXII (rFXII wt) (Fig. 2b). In addition to the heavy chain, the light chain at 25 kDa (IV) was found in preparations of αFXIIa and rFXII W268R, but not in rFXII wt. Treatment of rFXII W268R with kaolin resulted in increased generation of the light-chain fragment. Besides the heavy and light chains at 50 kDa and 25 kDa, respectively, we found in the rFXII W268R preparation a third fragment with an apparent molecular weight of 60 kDa (reducing) that was absent in the αFXIIa preparation (Fig. 2a, b). Mass spectrometry analysis of excised gel fragments confirmed the autoproteolytic cleavage of rFXII W268R at residues AA353/354, which yields in FXIIa. In addition, analysis of the 60- kDa fragment revealed cleavage of rFXII W268R after AA447, which is part of the protease domain and facilitated by kaolin treatment (Fig. 2c). In line with these observations which suggest autoactivation of FXII W268R, we found high spontaneous activity of FXIIa in the plasma of affected family members (Fig. 2d) as well as in the supernatant of FXII W268R-expressing HEK293 cells, but not in the plasma of healthy family members or wt-FXII expressing HEK293 cells (Fig. 2d, e). Interestingly, overall plasma levels of FXII were comparable with healthy controls (Supplementary Fig. 1), indicating that the *p.W268R* mutation only affects the probability of spontaneous FXII activation, but not its expression.

**Fig. 3:**
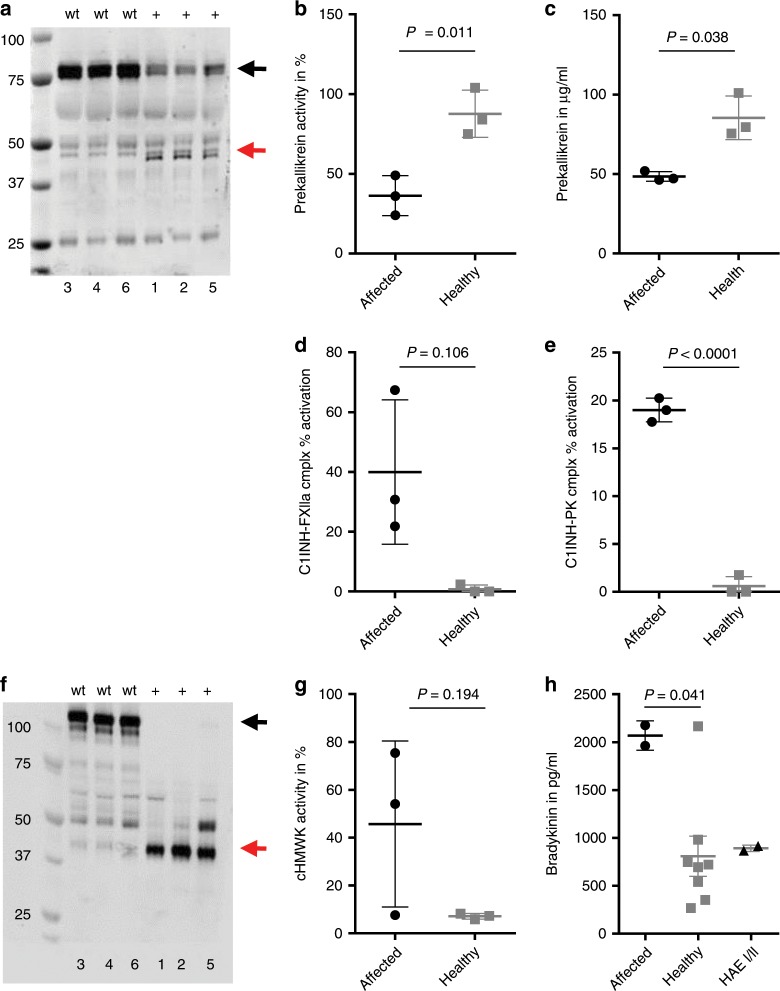
FXII W268R activates the kallikrein–kinin pathway. **a** Citrate plasma of affected (*n* = 3) and healthy family members (*n* = 3) was immunoblotted for prekallikrein (PK) showing substantial cleavage of PK (black arrow) to kallikrein (red arrow), *n* = 2 technical replicates. This corresponds to reduced PK activity (**b**) and plasma levels (**c**) in affected individuals (*n* = 3 affected vs. *n* = 3 healthy). **d** FXIIa-C1-inhibitor (FXIIa-C1-INH) and **e**) PK-C1-INH complexes in patient plasma (*n* = 3 affected vs. *n* = 3 healthy). **f** Citrate plasma of affected (*n* = 3) and healthy family members (*n* = 3) was immunoblotted for high-molecular-weight kininogen (HMWK) showing its complete degradation (black arrow) to cleaved HMWK (cHMWK) (red arrow), *n* = 2 technical replicates. Correspondingly, increased cHMWK activity (**g**) and bradykinin plasma levels (**h**) (*n* = 3 affected vs. *n* = 3 healthy; *n* = 2 affected vs. *n* = 7 healthy and *n* = 2 HAE I/II) are detectable in the plasma of affected patients. PK and cHMWK activity were measured by chromogenic assays, PK and bradykinin plasma levels were assessed by ELISA. Graphs show individual data points and the mean ± s.e.m. Statistical significance is indicated by unpaired Student’s *t* test with Welch’s correction (**b**, **c**, **d**, **e**, **g**) or one-way ANOVA with Sidak’s multiple comparisons test (**h**). Source data are provided as a source data file.

We next assessed mediators of the kallikrein–kinin pathway. High FXIIa activity was associated with pronounced plasma prekallikrein (PK) cleavage and kallikrein formation (Fig. 3a) similar to what is found in activated recombinant PK preparations (Supplementary Fig. 2). In line with these findings, we noticed reduced PK activity and plasma levels in affected patients (Fig. 3b, c). FXII-mediated contact system activation is counterbalanced by C1-INH to prevent continuous kallikrein and bradykinin formation. Thus, our observation of increased FXIIa-C1-INH and PK-C1-INH complexes in FACAS plasma reflected an imbalanced and constitutively active contact system (Fig. 3d, e). Kallikrein cleaves high-molecular-weight kininogen (HMWK) to release bradykinin, a potent but short-lived vasoactive mediator acting on bradykinin B2 receptors. Since bradykinin becomes rapidly degraded by kininases^16^, cleaved HMWK (cHMWK) is used as a surrogate marker of plasma bradykinin formation^17^. Interestingly, we found complete degradation of HMWK in the immunoblot of all tested FACAS patients (Fig. 3f). Correspondingly, the activity of cHMWK was clearly upregulated in patients vs. controls (Fig. 3g). Of interest, bradykinin plasma levels which were available from two FACAS patients revealed higher values compared with healthy controls (Fig. 3h).

**Fig. 4:**
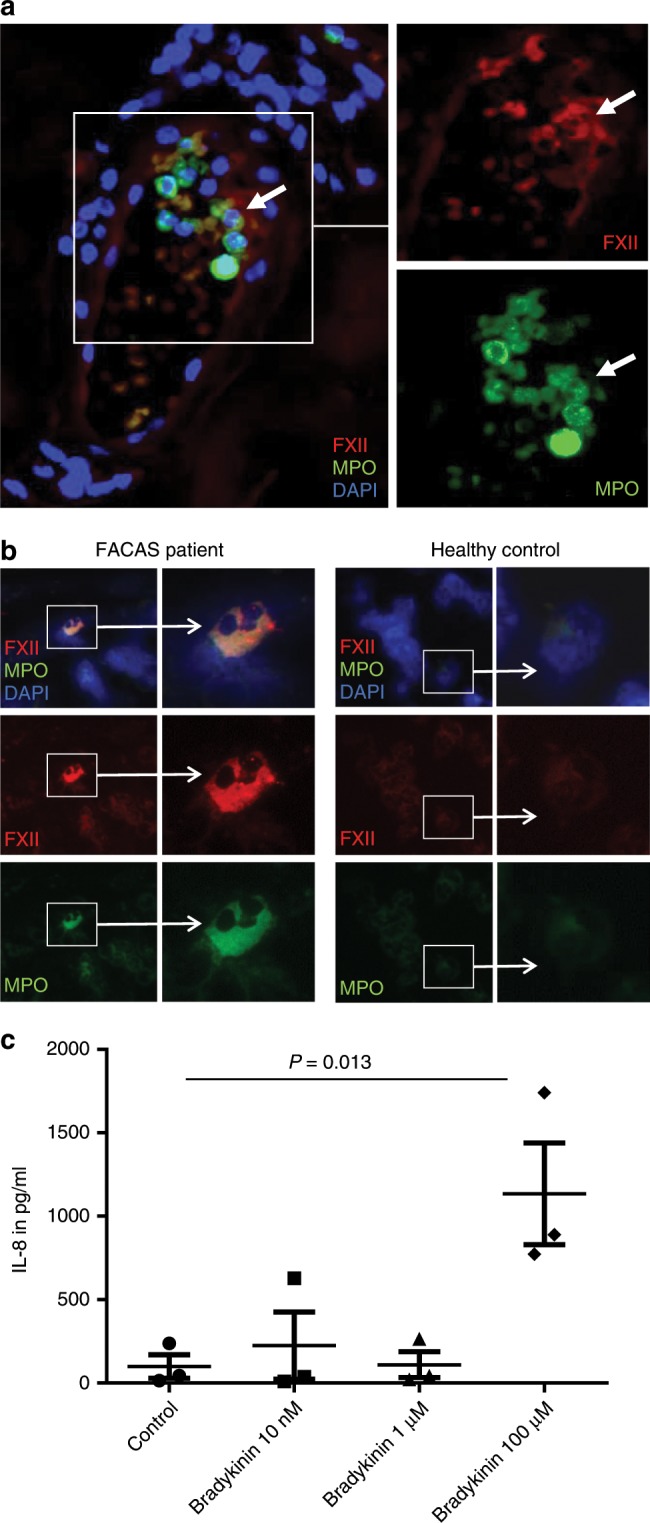
FXII is expressed by skin and blood neutrophils in FACAS. **a** Representative image from serial sections of the lesional skin of FACAS patient ca. 1 h after cold exposure. FXII immunoreactivity is restricted to neutrophils at perivascular sites and within blood vessels (white arrows), *n* = 2 technical replicates. **b** Exemplary images from cytospins isolated from the peripheral blood of FACAS patients and healthy control subjects. Only single FACAS neutrophils (0.72%), but not healthy control neutrophils (0%), present with double-positive cytoplasmatic staining for FXII and MPO. Original magnification ×400, *n* = 3 technical replicates. **c** Peripheral blood mononuclear cells were isolated from healthy control subjects by density-gradient centrifugation and stimulated with increasing doses of bradykinin. IL-8 secretion was assessed by ELISA. Pooled data of *n* = 3 different donors, *n* = 2 technical replicates. The graph shows individual data points and the mean ± s.e.m. Statistical significance is indicated by one-way ANOVA with Sidak’s multiple comparisons. Source data are provided as a source data file.

To investigate the possibility of local FXII production and activation at sites of inflammation, we evaluated the lesional skin of a FACAS patient following cold exposure. Immunohistochemistry revealed FXII expression by intra- and perivascular neutrophils (but not macrophages; Supplementary Fig. 3) as demonstrated by cytoplasmatic co-staining with antibodies for myeloperoxidase (MPO) and α-FXIIa heavy chain (Fig. 4a). Moreover, we found FXII expression in single peripheral blood neutrophils purified from an affected patient, but not in healthy controls (Fig. 4b). Together, these findings could point toward local FXII production by neutrophils in the skin of FACAS patients. As monocytes and tissue macrophages are known to recruit neutrophils via IL-8 secretion^18,19^, we hypothesized that contact system mediators might induce the attraction of neutrophils. Accordingly, we found dose-dependent IL-8 release from monocytes treated with increasing doses of bradykinin (Fig. 4c).

**Fig. 5:**
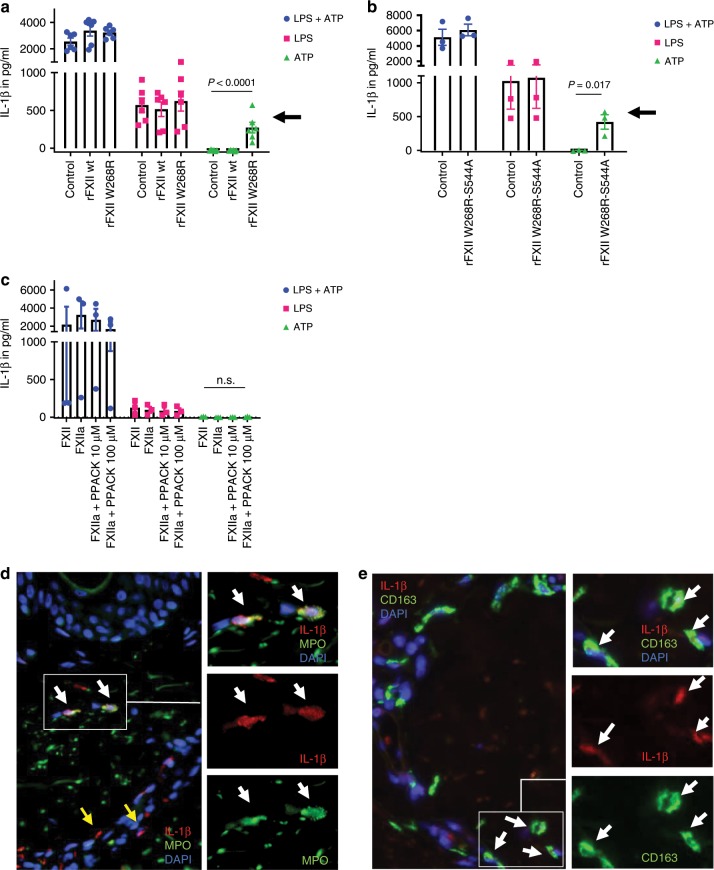
FXII W268R induces and increases IL-1β secretion and expression. **a**–**c** Peripheral blood mononuclear cells were isolated from healthy control subjects by density-gradient centrifugation. Interleukin-1β (IL-1β) secretion requires a two-step process: (i) transcriptional upregulation of pro-IL-1β via toll-like receptors and (ii) conversion of pro-IL-1β into its active form by inflammasome activation. Cells were pre-stimulated with toll-like receptor agonist lipopolysaccharide (LPS), and nucleotide binding like receptor protein 3 (NLRP3) inflammasome activation was induced by the danger molecule ATP (LPS + ATP = blue symbols). Red symbols denote LPS-stimulated cells only, and green symbols represent ATP-activated cells without priming by LPS. Experiments were performed in the presence of FXII-depleted plasma (control) and addition of recombinant FXII wild-type (rFXII wt) and W268R (rFXII W268R) protein (**a**) or rFXII W268R-S544A (**b**). Experiments in (**c**) were conducted with purified FXII, FXIIa, and different doses of the small peptide-based inhibitor PPACK. Black arrows indicate priming effect by the mutant rFXII W268R (**a**) and the active site incapacitated rFXII W268R-S544A (**b**), which was absent in rFXII wt and control. Also, the presence of FXIIa (with or without PPACK) did not induce changes in IL-1β release (**c**). IL-1β secretion was assessed by ELISA. Pooled data of *n* = 3 different (**a**) or *n* = 2 (**b**, **c**) experiments. Bar graphs show individual data points and the mean ± s.e.m. Statistical significance in is indicated by one-way ANOVA (**a**, **c**) with Tukey’s multiple comparisons test for ATP between the groups control vs. rFXII W268R and rFXII wt vs. rFXII W268R (**a**) or unpaired Student’s *t* test (**b**). **d, e** Representative image from serial sections of the lesional skin from FACAS patient following cold exposure exhibits IL-1β immunoreactivity. IL-1β expression is pronounced around blood vessels (yellow arrows) and co-localizes with neutrophils (**d**) and macrophages (**e**) (white arrows). Original magnification ×400, *n* = 2 technical replicates. Source data are provided as a source data file.

FXIIa and bradykinin were previously shown to induce IL-1β secretion from human monocytes and macrophages^20,21^. Assuming that FXIIa and/or consecutive mediators like bradykinin affect the IL-1β-related phenotype in FACAS, we investigated IL-1β responses in patient samples. Basal IL-1β secretion from patient PBMCs was negligible and comparable with healthy control PBMCs (Supplementary Fig. 4). However, exposure of healthy donor PBMCs to recombinant FXII proteins (wild-type and W268R) suggested an IL-1β priming effect of the FXII W268R mutant protein but not wild-type protein when co-stimulated with the NLRP3 activator ATP (without LPS) (Fig. 5a). To investigate whether the IL-1β priming effect was mediated via FXIIa activity, we assessed the potential of recombinant FXII W268R with an S544A inhibitory mutation^22^ (rFXII W268R-S544A) and αFXIIa to induce IL-1β release in PBMCs. Moreover, we blocked protease activity with the small peptide-based inhibitor PPACK (Phe-Pro-Arg-chloro-methylketone). Remarkably, the incapacitated S544A mutant protein showed a similar IL-1β priming effect as the active rFXII W268R (Fig. 5b). In contrast, the priming effect on IL-1β synthesis was absent in PBMCs treated with αFXIIa (Fig. 5c). Also, in LPS-primed and ATP-activated PBMCs, addition of FXIIa or FXIIa-PPACK did not induce significant changes on IL-1β release (Fig. 5c). Together, these results demonstrate that the priming effect on IL-1β synthesis is not linked to FXIIa activity.

When analyzing biopsies from lesional FACAS skin, we observed pronounced IL-1β expression (Fig. 5d, e). Positive IL-1β staining co-localized with perivascular neutrophils and macrophages, suggesting these cells as the primary source for IL-1β in the skin.

### Clinical efficacy of bradykinin B2 receptor antagonism

**Fig. 6:**
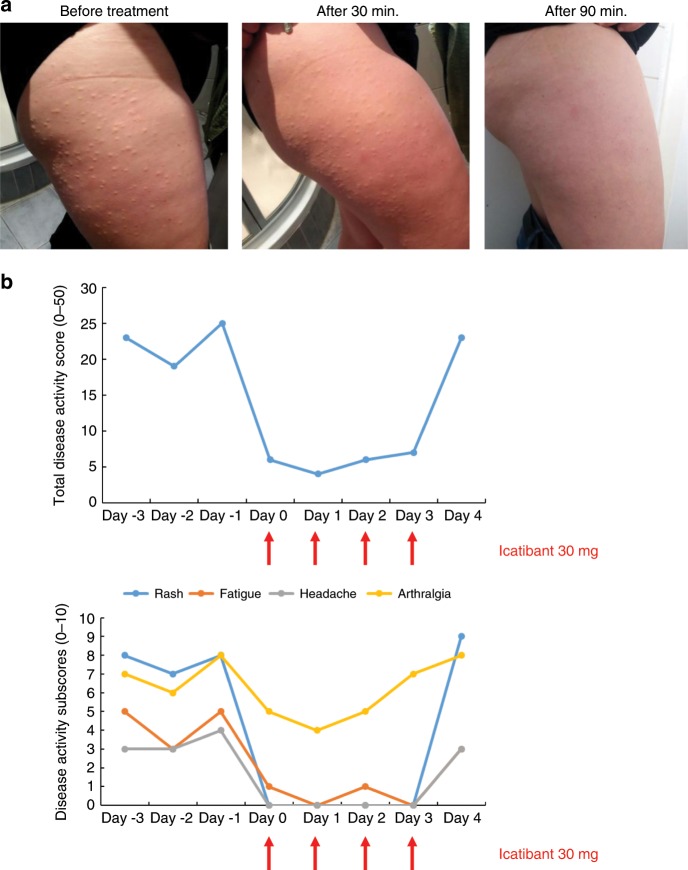
The bradykinin B2 receptor antagonist icatibant resolves FACAS signs and symptoms. During winter with continuous skin symptoms, the index patient administered icatibant 30 mg. **a** Urticarial rash completely resolved within 90 min post icatibant application (from the left to right). **b** Total and individual symptoms reported and assessed daily over a time period of 8 days by the patient using a standardized daily health assessment form covering the main symptoms rash, fatigue, headache, arthralgia, and fever/chills. The total score ranges from 0 = no symptoms to 50 = maximum of symptoms. Each subscore can range from 0 = no symptoms to 10 = maximum of symptoms. The subscores do not show fever/chills as these symptoms were not present at that time. Icatibant was given on 4 consecutive days (red arrows).

Based on the collected evidence for excessive contact system activation in FACAS, we initiated a short treatment course over 4 consecutive days with the bradykinin B2 receptor antagonist icatibant in the index patient (patient #1). Individual symptoms were assessed by a patient-reported standardized daily health assessment form that was adapted from a validated tool for CAPS^23^. Within 30 min after the first administration of 30 mg icatibant, the generalized urticarial rash and systemic symptoms improved. The maximum effect (absence of rash and headache, largely improved fatigue) occurred after 90 min and lasted for up to 12 h. Icatibant treatment was initiated during the winter and tolerated well. It completely abrogated skin and systemic symptoms while staying outside for several hours at temperatures below 0 °C. Only arthralgia was still mildly present. Complete relapse of symptoms occurred within 24 h after drug withdrawal (Fig. 6).

## Discussion

We report a dominantly inherited cold-induced urticarial autoinflammatory disease in a four-generation family that is associated with a *c.T859A* mutation in *F12* leading to an amino acid exchange of tryptophan by an arginine (*p.W268R*). Functional analyses of the mutant protein revealed high susceptibility to autocatalytic cleavage after residue AA353, which is known to be associated with FXIIa formation. This is paralleled by constitutive activation of the *p.W268R* variant of FXII resulting in an overactive contact system and release of potent inflammatory cytokines such as IL-1β. The relevance of bradykinin as a critical mediator is underlined by the immediate clinical response to treatment with the bradykinin B2 receptor antagonist icatibant. Skin and systemic symptoms associated with IL-1β-mediated inflammation in FACAS are reminiscent of the clinical phenotype of CAPS. Its underlying mechanisms, however, are different and share signaling cascades of kallikrein–kinin activation with HAE.

FXII is primarily produced and secreted by hepatocytes. It circulates as a single-chain inactive zymogen in the plasma and converts into FXIIa (composed of an N-terminal heavy chain and a C-terminal light chain) upon contact with negatively charged surfaces such as polyphosphates released by platelets or by limited proteolysis^15^. In the pathogenesis of FXII-HAE, defective glycosylation augments contact-mediated FXII autoactivation, which enhances bradykinin formation via the kallikrein–kinin system^24^. Our data, showing partially cleaved and active FXII in patient plasma as well as enzymatic activity of recombinant W268R in cell culture supernatants, suggest spontaneous, intracellular conversion of FXII into FXIIa in FACAS. In contrast, spontaneous FXII cleavage was absent in FXII-HAE plasma, which is in line with previous reports showing the need for a plasmatic trigger to cleave and activate FXII in HAE^25^.

The novel mutation induces an exchange of tryptophan to arginine (*W268R*) inside the kringle domain of FXII. Kringles are common in members of the coagulation cascade and are thought to play essential roles in binding target molecules/structures, such as membranes, other proteins, or phospholipids, and in the regulation of proteolytic activity^26,27^. Our observation of fragmented rFXII-*W268R* that is cleaved after residue AA353 argues for increased autoactivation and may explain the high spontaneous FXIIa activity observed in plasma and supernatant of HEK293 cells transfected with rFXII-W268R. These findings differ from recent studies in FXII-HAE with p.*T309R* mutation. This variant was demonstrated to be enzymatically truncated after residue AA309 resulting in the loss of the heavy-chain fragment and dysregulated PK activation^28^. Our data do not support truncation of *W268R* after residue AA268. In contrast, we observed additional cleavage after AA447 in the recombinant protein. However, this was not detected in plasma of the affected patients, suggesting that this might be the result of artificial expression in HEK293 cells. From our data, we conclude that high susceptibility to cleavage at the regular cleavage site at AA353/354 leads to continuously increased FXIIa activity. In addition, the exchange of the aromatic and lipophilic tryptophan to the hydrophilic and, at physiological pH, positively charged arginine may foster binding of mutant FXII to negatively charged surfaces, thereby further enhancing its activation state leading to an exhaustion of the contact system with fully cleaved PK and HMWK, and constantly raised bradykinin levels in the patient plasma.

**Fig. 7:**
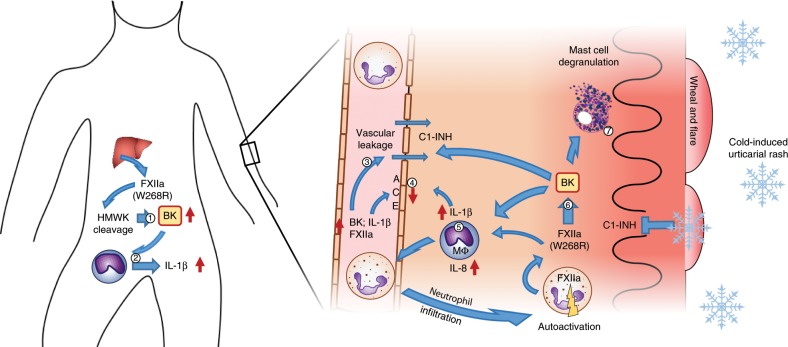
Proposed pathomechanism underlying FXII W268R mutation in FACAS. Liver-derived and pre-activated FXII induces plasma prekallikrein cleavage, HMWK cleavage leading to constantly raised bradykinin (BK) levels in the patient plasma (1) and increased permeability of the vasculature (3). BK may also induce IL-1β release from monocytes and tissue macrophages (Mɸ) leading to the autoinflammatory phenotype observed in FACAS patients (2), (5). IL-1β may further induce BK accumulation by upregulation of its receptors and downregulation of its inactivating enzyme ACE (4). In the skin, BK activates Mɸ to produce IL-8 and IL-1β, recruiting neutrophils into the tissue (5). These neutrophils may act as a source of auto-activated FXIIa in the skin, which leads to further contact system activation and BK generation (6). Under normal conditions, C1-Inhibitor can control BK generation, however, upon cold exposure inhibitory capacity may drop leading to a local increase in BK. Activation of neutrophils may occur via a paracrine feedback loop involving plasma kallikrein and HMWK. In addition, BK may activate skin mast cells to release heparin and polyphosphates (7), which supports further activation of FXII.

Neutrophil-dominated dermal infiltrates are known from urticarial autoinflammatory diseases Schnitzler’s syndrome and CAPS^29^. These are classified as neutrophilic urticarial dermatosis as opposed to the rather sparse lympho-histiocytic infiltrates in lesional skin of common urticaria. Their role, however, remains ill-defined. Interestingly, peripheral blood neutrophils were previously shown to produce zymogen FXII, which functionally differs from hepatic FXII and contributes to neutrophil trafficking via cell adhesion, migration, and release of extracellular DNA at sites of inflammation^30^. Conceivably, the generalized urticarial rash in FACAS could be mediated via neutrophil-derived and pre-activated FXII at the sites of inflammation as suggested by the cytoplasmatic expression of αFXIIa in the skin and peripheral blood neutrophils. IL-8 is known to induce neutrophil recruitment^19^. Supported by our data, plasma-derived BK may induce IL-8 production by skin macrophages to recruit neutrophils (Fig. 7). We hypothesize that upregulated FXII expression in FACAS neutrophils may be mediated via a paracrine feedback loop involving contact system components such as plasma kallikrein and HMWK. These were both shown to activate (and in case of HMWK also bind to) neutrophils^31,32^. Thus, the high degree of contact system activation could be the reason why FXII expression was restricted to FACAS and not observed in healthy control neutrophils.

The sensitivity to cold as a trigger and amplifier of clinical symptoms in FACAS is known from autoinflammatory diseases such as CAPS. Here, cold was proposed to escalate constitutive inflammasome activity via increased production of reactive oxygen species induced by cellular stress to temperature shifts^33^. In FACAS, however, inflammasomes of mononuclear cells are not constitutively activated (Supplementary Fig. 4), suggesting alternative mechanisms. Of interest, C1-INH activity was previously reported to be inhibited by cold as shown by complete plasma HMWK cleavage at 0 °C in vitro^34^. We assume that cold-induced decreases in tissue C1-INH activity may contribute to an imbalanced contact system in FACAS. Temperatures above a certain threshold (clinically around 20 °C) may protect from inflammatory symptoms because of adequate local C1-INH activity that counterbalances kallikrein activation. Based on this hypothesis, lower temperatures will reduce C1-INH activity leading to spontaneous but locally restricted activation of the kallikrein-kinin pathway and bradykinin production in the tissue, sufficient for wheal induction but not angioedema.

Bradykinin drives the inflammation in experimental models of osteoarthritis and neuroinflammation via upregulation of cytokines such as IL-1β^35,36^. Vice versa, IL-1β is known to augment the accumulation of bradykinin via upregulation of bradykinin receptors^37,38^ and activation of prekallikrein–HMWK complexes^39^. Also, IL-1β downregulates angiotensin-converting enzyme (ACE) expression in endothelial cells, resulting in less efficient bradykinin elimination^40^. Accordingly, elevated skin and systemic IL-1β levels in FACAS may amplify inflammatory responses via a positive feedback loop that induces further bradykinin production. Moreover, bradykinin was shown to activate and degranulate mast cells in vitro resulting in the release of heparin and polyphosphates, which may facilitate the local production of bradykinin via FXII activation^41,42^. Also, skin mast cells contribute to IL-1β-mediated inflammation in urticarial autoinflammatory diseases^43^, and their abundant expression and activation may promote the urticarial phenotype in FACAS patients (Fig. 7).

The clinical response to treatment with the IL-1 receptor antagonist anakinra mirrors the increased presence of IL-1β in FACAS tissue similar to other urticarial autoinflammatory diseases such as CAPS. Incomplete clinical improvement by anakinra may be explained by continuous bradykinin generation via complex feedback loops and possibly further cytokines such as TNFα, which are induced by bradykinin^44^ and not inhibited by IL-1 blockade. Of further interest, our findings suggest that the *p.W268R* variant can prime cells for IL-1β production via pattern-recognition receptors, without the need of an exogenous stimulus by pathogen-associated molecular patterns such as LPS. Surprisingly, the IL-1β priming effect is not linked to FXIIa activity as shown by the missing impact of αFXIIa on IL-1β secretion. Thus, we believe that conformational changes of FXII *p.W268R* result in the recognition as a danger signal that serves as toll-like receptor agonist and inducer of IL-1β release.

The prompt and striking clinical response to the bradykinin B2 receptor antagonist icatibant is in line with its efficacy in HAE patients and strongly supports our model of a bradykinin-mediated mechanism. Reasons for the persistence of mild arthralgia may be the short treatment duration of icatibant or the fact that bradykinin effects on joint inflammation are mediated via the B1 receptor rather than the B2^35^.

Limitations of our study include the small patient and sample sizes. Until now, there are no other families or individuals known with FACAS. Hence, it will be important to screen patients with similar phenotypes for the FXII *p.W268R* mutation in order to confirm our data. The mechanism by which cold induces inflammation as well as the role of neutrophils in FACAS need to be further evaluated. Also, the duration of treatment with anakinra and icatibant was only short due to side effects and limited efficacy (anakinra) or limited availability (icatibant). Data on long-term efficacy are still missing.

In conclusion, the pathomechanisms underlying this novel disease illustrate the critical role of fragmented and pre-activated FXII in driving skin and systemic inflammation as well as the close link between contact system activation and cytokine signaling. Therapeutic approaches in FACAS should aim at continuous control of inflammation in order to prevent long-term sequelae such as amyloidosis. Based on the short half-life of icatibant of few hours, inhibitors of contact activation including kallikrein antagonists and FXII antibodies are promising drug candidates of interest.

## Methods

### Patients

A total of eight family members, four of them affected and four non-affected, were analyzed. All of them provided written informed consent. This included the publication of individual data. For affected children, parents provided written informed consent, respectively. The authors affirm that human research participants provided informed consent for publication of the images in Figs. 1a, 6a. The study was approved by the local Charité Ethics Committee, Charité—Universitätsmedizin Berlin, Germany.

### Genetic studies

Genomic DNA was isolated from saliva samples of eight family members, and whole-exome sequencing (SureSelect human all exon v5.0, Agilent, Santa Clara, California) of three affected family members across three generations was performed and analyzed as described before in greater detail^45^. Briefly, genetic variants in the candidate genes were filtered to be present in all three sequenced individuals (in any zygosity) and to occur at <5% in 1000 Genomes database. DNA was available for follow-up Sanger sequencing from seven family members including all four affected individuals to determine consistency of variant segregation with disease. DNA exome capture was performed using the SureSelect human all exon v5 kit according to the manufacturer’s protocol and the library sequenced on the HiSeq 2000 platform (Illumina, San Diego, California), generating data sufficient to provide > 50× coverage across the exome. Read data were aligned using Novoalign v2.08 and duplicate reads marked using Picard v1.97. Variant calling was performed using Samtools v0.1.19 and variants annotated using Annovar (23rd August 2013 release) utilizing RefSeq gene definitions. Candidate genes were identified in HGMD release 2015.4 using the search term urticaria. Segregation analysis was performed on amplicons generated using primers forwards 5′-CTAACCTCCCGGGGTCTG-3′ and reverse 5′-CAGACCCCGGGAGGTTAG-3′ using standard techniques. Unidirectional Sanger sequencing was performed using the forward primer.

### Blood collection

Following standard procedures, nine volumes of blood were collected in one volume of sodium citrate, (3.2% w/v). Samples were centrifuged twice at 2000×*g* for 10 min. Plasma from FACAS-affected family members and from healthy donors was stored in aliquots at −80 °C until use. For PBMC stimulation assays, blood from FACAS-affected family members or healthy control donors was sampled into lithium heparin tubes and processed as explained below. Citrate and heparin tubes were from Greiner Bio One (Kremsmünster, Austria). For plasma bradykinin level assessment, blood was sampled into SCAT-169-4.5/5 protease inhibitor blood collection tubes (Haematologic Technologies, Essex Junction, Vermont), centrifuged at 2000 × *g* for 10 min and stored as described above.

### Western blot

Frozen plasma of affected and healthy individuals was thawed on ice, diluted 1:10 (FXII), 1:20 (HMWK), or 1:40 (PK) in sample buffer (15.5% glycerol, 96.8 mM Tris-HCl, 3.1% SDS, and 0.003% bromophenol blue, 25 mM DTT (Sigma-Aldrich, St. Louis, Missouri)), boiled for 5–10 min and briefly chilled on ice. Recombinant constructs were treated similarly. Approximately 50–100 ng total protein of recombinant proteins or 5 µl of plasma sample were loaded and separated on 10% Tris/glycine or Bolt 4–12% Bis-Tris Plus Gels (Merck-Millipore) and separated at 200 V (running buffer 190 mM glycine, 25 mM Tris, 0.1% SDS) or 165 V (MOPS running buffer (Merck-Millipore, Amsterdam, The Netherlands)), respectively, for 1 h. The gels were washed for 5 min in blotting buffer (14.4 g/l glycine, 3.03 g/l, Tris-HCl, 20% ethanol) and Whatman Protran nitrocellulose membranes (BA85, pore size 0.45 µm (Sigma-Aldrich)) were activated in blotting buffer for 5 min as well. Proteins were transferred onto nitrocellulose membranes with the TransBlot Turbo (BIORAD, Hercules, CA) for 30 min at 25 V and 1 A in 190 mM glycin, 25 mM Tris, 15% methanol or onto Immobilon-FL membranes (Merck-Millipore) at 125 V for 55 min in blotting buffer. The membranes were blocked for 1 h using Odyssey Blocking Buffer (LI-COR, Hamburg, Germany) diluted 1:1 with Tris-buffered saline (TBS; 50 mM Tris-HCl and 150 mM NaCl, pH 7.4). PK, HMWK and FXII were detected with affinity-purified polyclonal antibodies at 1:4000 (CL20090A, CL20027AP and CL20055AP, respectively, Cedarlane, Burlington, Canada) by overnight incubation at 4 °C. Membranes were washed with TBS, 0.05% Tween-20, and probed with Donkey-anti-goat-IgG DyLight800 conjugate (C30530-01, LI-COR) at 1:40,000 or Alexa Fluor 680 donkey anti-sheep IgG 1:5000 (A-21102, Thermo Fisher Scientific, Waltham, MA) for 1 h at room temperature. Membranes were washed again with TBS, 0.05% Tween-20, and images were developed using the Fusion FX (Vilber Lourmat, Eberhardzell, Germany) or a near-infrared Odyssey scanner (LI-COR) with excitation/emission at 640 nm/680 nm or 740 nm/800 nm.

### Mass spectrometry

FXII proteins (WT and W268R, full-length and cleavage products) planned to be analyzed by mass spectrometry were applied to 10% SDS-PAGE as described above (1 µg/lane), stained overnight at 4 °C using Instant Blue (ISB1L, Expedeon San Diego, California) and destained for ≥1 h at ambient with destaining solution (10% acetic acid, 20% ethanol). Defined bands were cut by scalpel, and sent for mass spectrometry at BIH Proteomics (Max Delbrück Center, D-13125 Berlin). Tryptic digest was performed in-gel on defined bands (Fig. 2b I, II, III, IV). Each band was cut into small pieces, washed with 50 mM ABC (ammonium bicarbonate) and 50% ethanol/50 mM ABC buffer, dehydrated with 100% Ethanol and vacuum dried, incubated for 45 min with 10 mM DTT at 56 °C, followed by 45 min incubation with 55 mM chloroacetamide (Merck-Millipore). After one round of 50 mM ABC and 50% ethanol/50 mM ABC buffer wash, gel pieces were dehydrated with 100% ethanol, dried and each sample was incubated with 1 µg trypsin (sequence-grade modified, V5111, Promega, Madison, Wisconsin) in 50 mM ABC buffer overnight at 37 °C. After acidifying, the samples with formic acid (2% final concentration), peptides were extracted with 30% ethanol/50 mM ABC followed by 100% acetonitrile. The supernatants were combined and dried down. The resulting peptide pellet was dissolved in 3% acetonitrile/0.1% formic acid and analyzed by mass spectrometry. For mass spectrometric measurements, the peptide mixture was separated by reversed-phase chromatography using an EasyLC 1200 system (Thermo Fisher Scientific) on in-house-manufactured 20-cm fritless silica microcolumns packed with ReproSil C18 resin (inner diameter 75 µm, Dr. Maisch GmbH, Ammerbuch-Entringen, Germany). Peptides were separated using an 8–60% acetonitrile gradient (44 min length) at a nanoflow rate of 250 nl/min. Eluting peptides were directly ionized by electrospray ionization and analyzed on a Q Exactive HF-X (Thermo Fisher Scientific). Mass spectrometry was performed in data-dependent positive mode with one full scan (m/z range = 350–1800; *R* = 60000; target value: 3 × 10^6^; maximum injection time = 45 ms). The Top 20 most intense ions with a charge state between 2 and 7 were selected (*R* = 45000, target value = 1 × 10^5^; isolation window = 1.3 m/z; maximum injection time = 86 ms). Dynamic exclusion for selected precursor ions was set to 20 s. Data analysis was performed using MaxQuant software package (version 1.6.0.1^46^). The internal Andromeda search engine was used to search MS^2^ spectra against human coagulation factor XII wild-type and W268R mutant sequence containing forward and reverse sequences. A database for contaminants was included. The search included variable modifications of methionine oxidation, N-terminal acetylation, deamidation (N and Q), and the fixed modification of carbamidomethyl cysteine. Minimal peptide length was set to six amino acids, digestion parameter was semitryptic. The FDR was set to 0.01 for peptide, protein, and site identifications. To filter for confidently identified peptides, a minimum of two MS counts and valid value in both replicates for at least one sample group were required. Intensity values were log2 transformed. Mass spectrometry was performed twice; *n* = 2 duplicates were assessed for each band.

### Factor XII protein expression and purification

The eukaryotic expression vector pcDNA6/V5-His A (Thermo Fisher Scientific) was modified by insertion of a murine Igκ secretion signal and two strep-tags through the HindIII and EcoRI digestion sites (called pSM2). cDNA of the F12 gene was kindly donated by Dr F. Citarella and matches the National Center for Biotechnology Information reference sequence (NM_000505.3), except for the silent mutation c.nt.1140 T > C (P460P, mature protein numbering). A sequence coding for a tobacco etch virus (TEV) protease cleavage site was inserted from the F12 sequence by using PCR with the Phusion Polymerase (Thermo Fisher Scientific). FXII-W268R was developed through two-step assembly of three double-stranded DNA segments (annotated as N, M and C, respectively; Primer sequences in Table 2). Fragment N with the desired mutation (T859A) was ordered as double-stranded DNA fragments from Integrated DNA technologies (Coralville, Iowa) and ligated into the pJET1.2/blunt vector (Thermo Fisher Scientific) according to the manufacturer instructions and amplified through PCR. Fragments M and C were amplified by PCR from FXII-WT cDNA in pSM2. First, the N and M segments were digested with XmaI and subsequently, fused by ligation with T4 ligase (Thermo Fisher Scientific). The fused product (NM) was amplified via PCR. Hereafter, the NM and C segments were digested with BsaI and fused together by ligation with T4 ligase. The assembled cDNA product was ligated into the pJET1.2/blunt vector according to the manufacturer’s instructions. Inserts were digested with EcoRI and NotI and ligated into the EcoRI and NotI-restricted pSM2 vector. All restrictases were purchased from New England Biolabs (Ipswich, MA). Sequences were verified by Sanger sequencing (Macrogen Europe, Amsterdam, The Netherlands) prior to transfection. HEK293F (LGC Standards GmbH, Wesel, Germany) cells were transfected using 293fectin (Thermo Fisher Scientific) and cultured in Gibco FreeStyle 293 Expression Medium (Thermo Fisher Scientific) at a concentration of 1 × 10^6^ cells/ml in 2 ml. Four days after transfection, the cell suspension was centrifuged and 500 × *g*, and the supernatant was collected and stored until use at −20 °C. For protein purification, transfection was repeated in a volume of 1-l cell suspension, and culture medium was harvested after 1 week. FXII-W268R was purified via a strep-tag using Strep-Tactin Sepharose beads (IBA-Life Sciences, Goettingen, Germany) and dialyzed overnight at 4 °C against 4 mM sodium acetate–HCl with 150 mM NaCl (pH 5.5) using a Spectra/Por dialysis membrane (molecular weight cutoff 3.5 kDa, Spectrum Labs, Rancho Dominguez, California) that was preincubated with polybrene (Sigma-Aldrich).

**Table 2:** Primer sequences for FXII genetic constructs.

N-Frag_For	GGAGAATTCGAAAACCTGTATTTTCAGTCTATTCCACC
N-Frag_Rev	GCTCCCGGGGTCTGGGACTGAGGCGGGG
M-Frag_For	GACCCCGGGAGCCTTGCCGGCGAAGCG
M-Frag_Rev	CGTGGTCTCGGAGGGTCGCGCGGCGCCGCTTG
C-Frag_For	GACCCTCCGAGACCACGCTCTGCCAGG
C-Frag_Rev	CGAGCGGCCGCTCATCAGGAAACGGTGTGCTCCCG

### Chromogenic assays

Chromogenic substrate H-D-Pro-Phe-Arg-pNA (0.5 mM, Bachem, Bubendorf, Switzerland) was used to detect FXII activity from recombinant constructs in culture medium. Ninety-six-well Costar plates (Corning, New York) were blocked with HBS (10 mM HEPES (VWR International, Amsterdam, The Netherlands), 150 mM NaCl, 1 mM MgSO_4_, 5 mM KCl, pH = 7.4) containing 0.1% BSA and subsequently, 40 -µl culture medium supernatant containing rFXII was added followed by 10 µl of substrate. Substrate conversion was immediately read out at 405 nm at 37 °C. FXII activity in plasma was assessed using the Chromogenic Assay for Factor XIIa-like Activity (COA0088, CoaChrom Diagnostica, Maria Enzersdorf, Austria) as specified by the manufacturer. Absorbance signals were measured in the VIKTOR X5 (PerkinElmer, Waltham, MA), and analyzed using GraphPad Prism 7 or Microsoft Excel 2016.

### Factor XII concentration ELISA

FXII concentration in plasma was assessed using the Matched-Pair Antibody Set ELISA of human Factor XII antigen (FXII-EIA, CoaChrom Diagnostica) as specified by the manufacturer. Absorbance signals were measured in the VIKTOR X5 (PerkinElmer, Waltham, MA), and analyzed using GraphPad Prism 7 or Microsoft Excel 2016.

### PK activity (routine lab) and PK ELISA

Prekallikrein activity was assessed by coagulometric assay in routine measurements at Labor Berlin (13353 Berlin, Germany). Prekallikrein plasma concentration was measured using the Prekallikrein and Kallikrein Human ELISA (ab171015, Abcam, Cambridge, UK) as specified by the manufacturer. Absorbance signals were measured in the VIKTOR X5 (PerkinElmer, Waltham, MA), and analyzed using GraphPad Prism 7.02 or Microsoft Excel 2016.

### Enzyme-C1-INH complexes ELISA

Methods on V_H_H (variable domain heavy-chain-only antibody) selection, production, and execution for this assay are described earlier by de Maat^25^. In brief, V_H_H 1B12 (5 µg/ml), a monoclonal V_H_H directed against complexed C1-INH, was immobilized on 96-well Maxisorp plates (Corning). Plasma samples were diluted in 10 mM HEPES, 150 mM NaCl, 1 mM MgSO_4_, 5 mM KCl, pH = 7.4 (HBS) supplemented with 0.1% (w/v) Tween-20 and 1% (w/v) skimmed milk powder (Sigma-Aldrich) and containing 200 µM Phe-Pro-Arg-chloromethylketone (PPACK, Haematologic Technologies). For reference, normal pooled plasma from healthy donors (NPP) activated with DXS (average molecular weight 500.000, Sigma-Aldrich) for 30 min as well as non-activated NPP were prepared in HBS containing PPACK and mixed to generate samples containing 0–100% DXS-activated sample. Samples were incubated on the plate for 1 h. Enzyme present in the captured complexes was detected with a biotinylated polyclonal V_H_H targeted against the βFXIIA (5 µg/ml, made and biotinylated in-house^47^) followed by streptavidin-poly-HRP 1:4000 (M2051, Sanquin Blood Supply, Amsterdam, The Netherlands) or a polyclonal sheep anti-PK IgG 1:2000 (SAPK-AP, Affinity Biologicals, Hamilton, Canada) followed by HRP-labeled rabbit anti-sheep IgG 1:4000 (P0163, Dako, Heverlee, Belgium). Finally, 3,3′,5,5′-tetramethyl-benzidine (TMB, Tebu Bio, Heerhugowaard, The Netherlands) was added, and substrate conversion was stopped after 5–10 min with H_2_SO_4_ (0.3 M), and absorbance was read out at 450 nm. GraphPad Prism 7.02 software was used to interpolate the data from the reference range.

### Cleaved kininogen activity ELISA

Methods on V_H_H selection, production, and execution for this assay are described more detailed by Hofman et al.^48^. In brief, patients samples were diluted 64-fold in DPBS (Thermo Fisher Scientific) supplemented with 0.1% (w/v) Tween-20 and 1% (w/v) skimmed milk powder and containing 200 µM PPACK and 0.5 µg/ml DXS to generate HMWK complexes. For reference^5^, min βFXIIa-activated NPP and non-activated NPP samples were prepared and mixed to generate samples with various amounts of cHMWK. After incubation, cHMWK was detected using a biotinylated monoclonal anti-HMWK V_H_H H4 (2 µg/ml, made and biotinylated in-house) and streptavidin-poly-HRP 1:2000. TMB substrate conversion was measured and analyzed as described for the enzyme-C1-INH complex assays.

### Bradykinin ELISA

Bradykinin levels in patient and healthy control plasma were assessed by ELISA (ADI-900-206, Enzo Life Sciences, Farmingdale, New York) as described by the manufacturer. Absorption signals were measured in the VIKTOR X5 and analyzed using GraphPad Prism 7.02 or Microsoft Excel 2016 against bradykinin standard curves.

### IL-1β and IL-8 ELISA

IL-1β/IL-8 secretion was assessed by ELISA (DuoSet, DY201/DY208-05, R&D Systems, Minneapolis, MN) as specified by the manufacturer. For greater precision, ELISAs were carried out at ¼ of the recommended volumes per well in black NUNC 384-well MaxiSorp plates (Thermo Fisher Scientific) for 4× replicate measurements per original 96-well cell culture plate well. In addition, instead of TMB, we developed the assay using luminol/H_2_O_2_ for increased sensitivity (Amersham ECL Prime, GE Healthcare, Chicago, IL). Luminescence signals were measured in the VIKTOR X5 and analyzed using GraphPad Prism 7.02 or Microsoft Excel 2016 against IL-1β/IL-8 standard curves.

### Immunofluorescence cytology/histology

Neutrophils from patient and healthy donor blood were isolated using the MACSxpress Neutrophil isolation Kit (130-104-434, Miltenyi Biotec, Bergisch Gladbach, Germany), as specified by the manufacturer. Isolated neutrophils were washed once with DPBS and ca. In total, 10^5^ cells were applied to cytospin. Briefly, cells were concentrated to dense spots by centrifugation onto SuperFrost Plus microscopy slides (Thermo Fisher Scientific) through perforated cytospin microtubes (Frötek-Kunststofftechnik GmbH, Osterode am Harz, Germany) and Heraeus perforated cytospin filter cards (Thermo Fisher Scientific) with subsequent fixation by acetone at −20 °C. Cytospins were encircled by PAP pen, blocked with serum-free protein block (S3022, Dako) for 10 min at RT, and incubated with primary antibody against FXII 1:100 (sc-376770, Santa Cruz Biotechnology, Dallas, TX) or IL-1β 1:100 (LS-C174781, LSBio, Seattle, WA) in 2% goat normal serum in TBS for 3 h at RT. After rinsing 3 × 5 min with TBS, goat anti-mouse IgG Alexa Fluor 594 conjugate (115-585-062, Jackson Immuno Research, West Grove, Pennsylvania) was applied 1:200 in 2% goat normal serum in TBS for 30 min in the dark, again with subsequent 3 × 5 min rinsing with TBS. In case of double stainings, primary antibody against MPO (MAB3174, R&D Systems) was incubated together with goat anti-mouse IgG Alexa Fluor 488 conjugate (115-545-146, Jackson Immuno Research) 1:100 and 1:200, respectively, in 2% goat normal serum in TBS for 1 h in a microtube in the dark. Free anti-mouse IgG was blocked with 4% final normal mouse serum for 1 h in the dark, and antibody–antibody–dye conjugate was applied to the cytospins and incubated overnight at 4 °C in the dark. After rinsing 3 × 5 min with TBS, slides were air-dried briefly, mounted using DAPI-Fluoromount G (Thermo Fisher Scientific), and air-dried in the dark for several hours before microscopy. Patient biopsy was fixed in 4% buffered formalin overnight at RT, and paraffinated using an automated histokinette (Shandon Citadel 2000 Tissue Processor, Thermo Fisher Scientific). In all, 5-µm skin tissue sections were applied to SuperFrost Plus slides, baked at 60 °C for 1 h and subsequently deparaffinated with 2 × 10 min xylene, 2 × 2 min 100% ethanol, 2 × 2 min 96% ethanol, 2 min 70% ethanol, and 3 min ddH_2_O. Antigens were retrieved by incubation at 95 °C in citrate buffer (1.8 mM citric acid, 8.6 mM Na_3_ citrate, pH = 6) in a humid atmosphere for 15 min. Tissue sections were rinsed in TBS, encircled with PAP pen, and stained as described above. Samples of liver tissue (kindly provided by Charité Dpt. of Pathology) served as positive control for FXII. Sections omitting the primary antibody served as negative controls. Slides were analyzed by fluorescence microscopy using a Zeiss Axioplan 2 with Axiovision software 4.8.1. Images of cytospins were further analyzed by ImageJ 1.52a.

### Immunohistochemistry

Paraffin sections (5 µm) of a FACAS patient skin sample and control skin were prepared as explained above, and processed for routine (hematoxylin/eosin) and immunohistological stainings. We used an immunohistochemistry protocol (streptavidin–biotin labeling) employing primary antibodies targeting MPO (MAB3174, R&D Systems) 1:400 overnight at 4 °C or CD163 (ab189915, Abcam) 1:400 overnight at 4 °C. After washing 3 × 5 min with TBS, REAL Detection System Alkaline Phosphatase/RED (K5005, Dako) for MPO staining or labeled polymer-HRP anti-rabbit (K4011, Dako) together with AEC + high sensitivity substrate chromogen ready-to-use (K3461, Dako) for CD163 staining were applied according to the manufacturer’s instructions, and subsequently analyzed by bright-field microscopy. Samples of tonsil tissue served as positive controls. Sections omitting the primary antibody served as negative controls.

### PBMC stimulation assays

Venous blood from patients and healthy donors was sampled into heparin tubes. In all, 15 ml of Ficoll-Paque solution (GE Healthcare) were pipetted into 50-ml Leucosep tubes (Greiner Bio One) and centrifuged at 1000 × *g* for 1 min. Up to 20 ml of blood sample per tube was added, filled upto 50 ml with DPBS and centrifuged for 10 min at 1000 × *g* with 33% acceleration and without breaks. The upper plasma layer was discarded, the PBMC layer was collected and filled into a new 50-ml tube. PBMCs were washed twice by adding ad 50 ml DPBS, centrifugation for 10 min at 350 × *g*, and resuspension in residual DPBS. After the washing steps, 10 ml of PBMC medium (RPMI 1640 containing 1% FBS (both from Biochrom, Berlin, Germany), 100 U/ml penicillin, 100 µg/ml streptomycin (HyClone 100x Pen/Strep, GE Healthcare)) was added, and cells were counted using trypan blue staining and hematocytometer. PBMC number was adjusted to 3 × 10^6^/ml, and 100 µl of the suspension were added per well to Falcon TC treated 96-well plates (Corning). After 2 h incubation at 37 °C and 5% CO_2_, the supernatant containing non-monocytes was discarded and to the adherent monocytes, 100 µl of containing 0.5 ng/ml LPS (*E. coli* R515, Enzo Life Sciences) or not was added for NF-κB priming. Monocytes were incubated for 20 h at 37 °C and 5% CO_2_, supernatant was discarded, and 20 µl per well of PBMC medium was added. In all, 20 µl of either bradykinin at stated final concentrations or FXII constructs (with or without stated final concentrations of PPACK) diluted in PBMC medium were added at a final concentration of 35 ng/µl, followed by incubation at 37 °C and 5% CO_2_ for 1 h. Subsequently, 10 µl of 10 mM ATP (Sigma Aldrich) diluted in PBMC medium were added to a final concentration of 2 mM for inflammasome activation, followed by another 1 h incubation at 37 °C and 5% CO_2_. Supernatants were collected and stored at −20 °C until analysis by ELISA.

### Statistical analysis

Sample sizes were limited due to patient availability. The number of samples (all assessed in duplicates) and experiments are displayed and listed in the figure legends. The use of statistical tests is indicated in the figure legends as well.

### Reporting summary

Further information on research design is available in the Nature Research Reporting Summary linked to this article.

## Supplementary information

Supplementary Information

Reporting Summary

## Data Availability

Source data for figure numbers 2a–e, 3a,c–h, 4b, c, 5a–c, and Supplementary Figs. 1, 2 and 4 are provided as a Source Data file (10.6084/m9.figshare.11117669)^49^. The mass spectrometry proteomics data have been deposited to the ProteomeXchange Consortium via the PRIDE [1] partner repository with the dataset identifier PXD016341. As the informed consent obtained from FACAS patients does not allow for public deposition of the sequencing data, the filtered WES sequencing data from patients #2, #5, and #7 can be communicated upon reasonable request to R.J.P. and S.E. All other relevant data are contained within the article and its Supplementary Information.
