# Supplementary material for: Cold-induced urticarial autoinflammatory syndrome related to factor XII activation
Source: Nat Commun. 2020 Jan 10;11:179. doi: 10.1038/s41467-019-13984-8 (PMC6954242; doi:10.1038/s41467-019-13984-8)
Supplement: Supplementary file 1 — Supplementary Information [file 41467_2019_13984_MOESM1_ESM.pdf]

Cold-induced urticarial autoinflammatory syndrome related to factor XII activation

Scheffel et al.

Supplementary Information

Table of Contents:

Supplementary Table 1

Supplementary Figures 1-4

Supplementary Table 1

Characteristics, triggers and clinical phenotype of different cold-induced urticarial disorders

	**FACAS**	**CAPS**	**FCAS 2**	**FCAS 3**	**PLAID**	**Acquired cold urticaria**
**Gene**	*F12*	*NLRP3*	*NLRP12*	*NLRC4*	*PLCG2*	-
**Disease triggers**	Generalized exposure to cold (humid) air	Generalized exposure to cold (humid) air; Stress, infections	Generalized exposure to cold (humid) air; Stress, infections	Generalized exposure to cold (humid) air; Stress, infections	Generalized exposure to cold air; Evaporative cooling of skin; Cold water immersion	Direct skin contact with cold air, liquids or solids
**Cold contact provocation tests (Ice cube/ TempTest®)**	Negative	Negative	Negative	Negative	Negative	Positive
**Characteristics of skin lesions**	Generalized wheals or erythematous papules/plaques resolving after hours	Generalized wheals or erythematous papules/plaques resolving after hours	Generalized wheals or erythematous papules/plaques resolving after hours	Generalized wheals or erythematous papules/plaques resolving after hours	Localized wheals resolving after minutes - hours; Chronic granulomatous skin lesions in few patients	Localized wheals/ angioedema at sites of cold contact resolving after minutes
**Skin sensations**	Pruritus, burning and pain	No or only mild pruritus, burning or pain	No or only mild pruritus, burning or pain	No or only mild pruritus, burning or pain	Pruritus	Pruritus
**Systemic/ associated disease**	Musculoskeletal involvement, chills/fever, headache, fatigue	Musculoskeletal involvement, chills/fever, headache, fatigue; Uveitis; Sensorineural hearing loss and aseptic meningitis in severe phenotypes MWS/NOMID	Musculoskeletal involvement, chills/fever, headache, fatigue	Musculoskeletal involvement, chills/fever, headache, fatigue; Gastrointestinal involvement; Macrophage activation syndrome in severe phenotypes	Autoimmune disease; Immunodeficiency; Type I allergies	None
**Disease onset and course**	After birth/infancy; Lifelong	After birth/infancy, rarely late-onset adulthood; Lifelong	After birth/infancy; Lifelong	After birth/infancy; Lifelong	After birth/infancy; Lifelong	Variable, any age; Spontaneous remission

FACAS = FXII-associated cold autoinflammatory syndrome; CAPS = Cryopyrin-associated periodic syndrome; FCAS = Familial cold autoinflammatory syndrome; MWS = Muckle-Wells syndrome; NOMID = Neonatal-onset multisystem inflammatory disease; PLAID = PLCG2-associated antibody deficiency and immune dysregulation

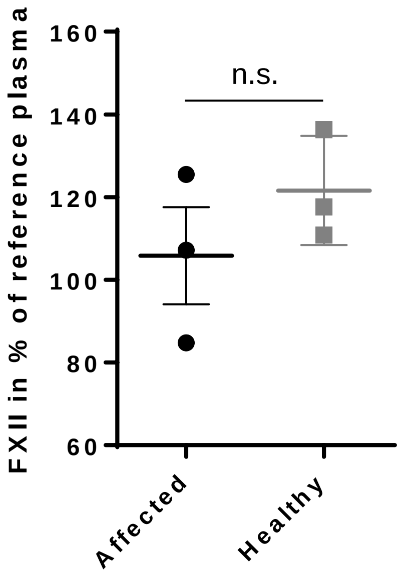

Supplementary Figure 1: FXII plasma levels in patients are comparable to healthy controls. FXII plasma levels from FACAS (black bars) and healthy control (grey bars) subjects (n=3 affected vs. n=3 healthy) were assessed by ELISA. Graph shows individual data points and the mean ±sem. Statistical significance was assessed by unpaired Student's t-test with Welch's correction. Source data are provided as a source data file.

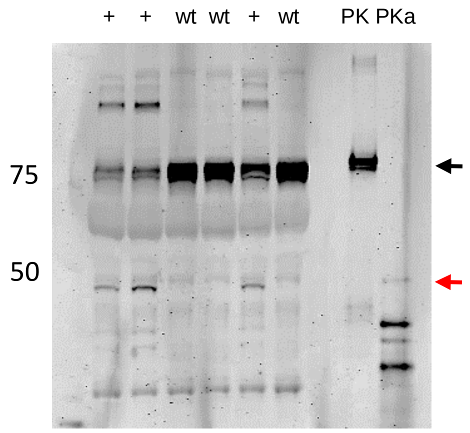

Supplementary Figure 2: Citrate plasma of affected (n=3) and healthy family members (n=3) was immunoblotted for prekallikrein (PK) showing substantial cleavage of PK (black arrow) and kallikrein (red arrow) formation when compared with activated recombinant PK, n=2 technical replicates. Source data are provided as a source data file.

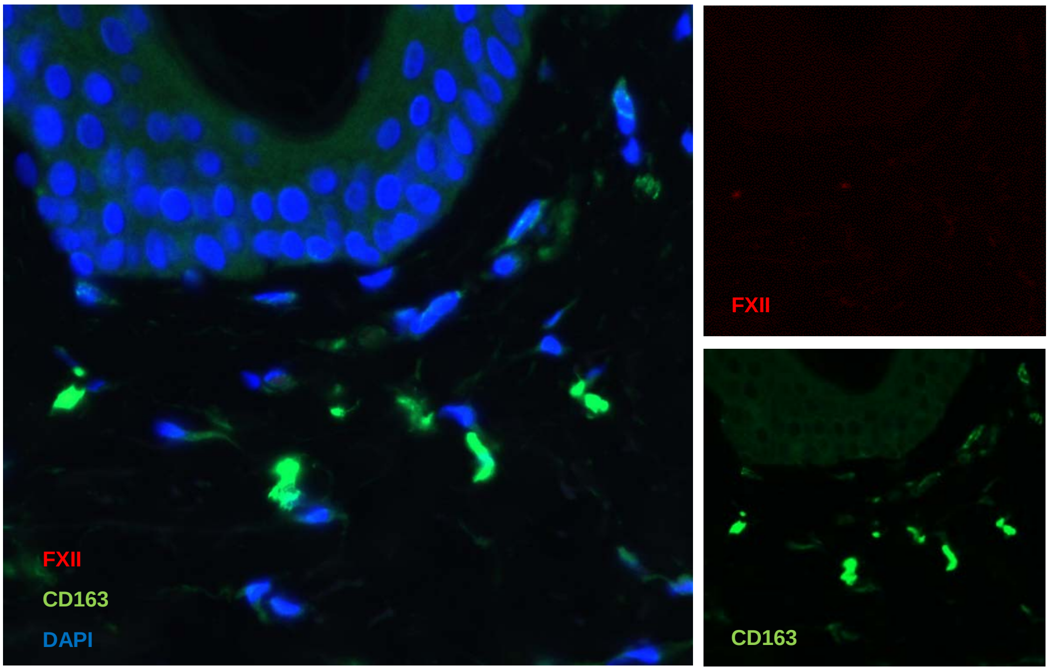

Supplementary Figure 3: FXII is not expressed by skin macrophages of FACAS patients. Lesional skin of a FACAS patient ca. 1h after cold exposure. FXII immunoreactivity does not co-localize with macrophages (green fluorescence). Original magnification 400x, n=2 technical replicates.

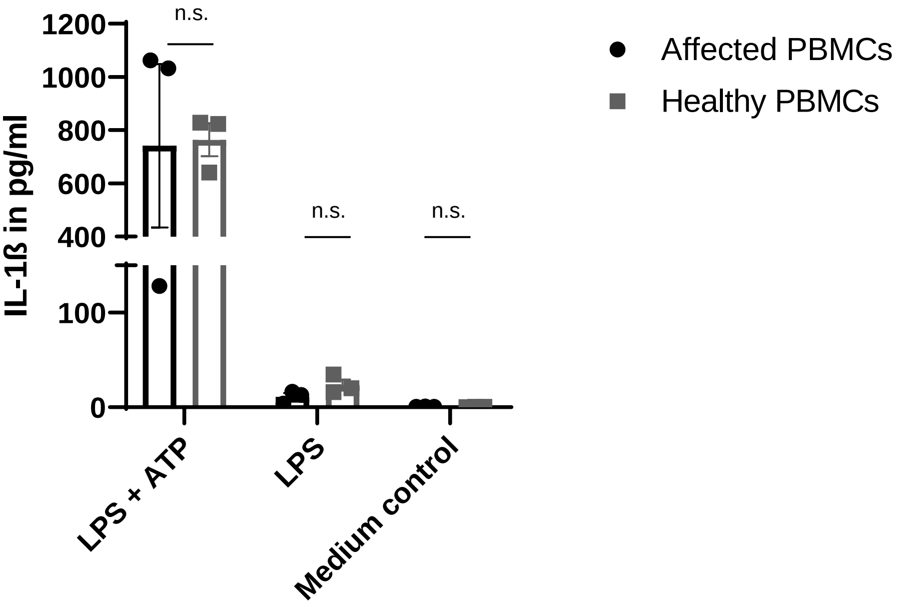

Supplementary Figure 4: Patient PBMCs show low basal IL-1ß secretion that is comparable to healthy controls. Peripheral blood mononuclear cells were isolated from FACAS (black bars) and healthy control (grey bars) subjects (n=3 affected vs. n=3 healthy) by density gradient centrifugation. Cells were pre-stimulated with LPS and activated by ATP, LPS-stimulated only and left untreated (medium ctrl). Bar graphs show individual data points and the mean ±sem. Statistical significance was assessed by unpaired Student's t-test with Welch's correction. Source data are provided as a source data file.
